# Evaluation of the anti-cancer potential of *Cedrus deodara* total lignans by inducing apoptosis of A549 cells

**DOI:** 10.1186/s12906-019-2682-6

**Published:** 2019-10-24

**Authors:** Xiaofeng Shi, Ruiqin Du, Junmin Zhang, Yanping Lei, Hongyun Guo

**Affiliations:** 1 Gansu Provincial Cancer Hospital, Lanzhou, 730050 China; 2 Gansu Provincial Academy of Medical Sciences, Lanzhou, 730050 China; 3 School of Pharmacy, Lanzhou University, Lanzhou, 730030 China

**Keywords:** Total lignans, *Cedrus deodara*, Total lignans, Anti-cancer, Apoptosis

## Abstract

**Background:**

*Cedrus deodara* (Roxb.) Loud (normally called as deodar), one out of four species in the genus *Cedrus*, exhibits widely biological activities. The *Cedrus deodara* total lignans from the pine needles (CTL) were extracted. The aim of the study was to investigate the anticancer potential of the CTL on A549 cell line.

**Methods:**

We extracted the CTL by ethanol and assessed the cytotoxicity by CCK-8 method. Cell cycle and apoptosis were detected by a FACS Verse Calibur flow cytometry.

**Results:**

The CTL were extracted by means of ethanol hot refluxing and the content of total lignans in CTL was about 55.77%. By the CCK-8 assays, CTL inhibited the growth of A549 cells in a dose-dependent fashion, with the IC_50_ values of 39.82 ± 1.74 μg/mL. CTL also inhibited the growth to a less extent in HeLa, HepG2, MKN28 and HT-29 cells.

**Conclusion:**

At low doses, the CTL effectively inhibited the growth of A549 cells. By comparison of IC_50_ values, we found that A549 cells might be more sensitive to the treatment with CTL. In addition, CTL were also able to increase the population of A549 cells in G2/M phase and the percentage of apoptotic A549 cells. CTL may have therapeutic potential in lung adenocarcinoma cancer by regulating cell cycle and apoptosis.

## Background

*Cedrus deodara* (Roxb.) Loud (*CD*), normally called as deodar, is one of *Cedrus* that was first described by Trew [1, 2], and is a species of cedar native to the Western Himalayas in Eastern Afghanistan, Northern Pakistan, South Western Tibet, North-Central India, and Western Nepal [2]. As one of the widely used traditional medicines, *CD* exhibits a variety of biological and pharmacological activities [1, 2]. The wood of *CD* has long been used to treat rheumatoid arthritis and inflammation in Indian [3, 4]. Again, *CD* has been described to display therapeutic effects in expelling wind, destroying parasites, removing dampness and relieving itches in the Dictionary of Chinese Crude Drugs. In addition, it has been widely utilized in Chinese drinks and has been recommended in the Ayurvedic system of medicine. Clinically, it is also far and wide used to alleviate arthralgia, sleeplessness, traumatic injury, eczema, ascariasis, and edema.

As our continuous efforts in discovering the pharmacological mechanism of *CD*, we have successfully extracted three kinds of compounds: lignans, terpenes and flavonoids. As is case for *CD,* these chemical compounds exhibit therapeutic effects against inflammation, pain, spasm, diabetes, Herpes simplex virus type-1 and cancer [5, 6]. Their antivirus and antibacterial effects have also been reported [7, 8]. In addition, it is reported that cedrin identified from *CD* protects PC12 cells against neurotoxicity induced by A*β*_1–42_ [9]. The chemical constituents of pine needles of *CD* were investigated in our lab. More than 40 pure compounds, including lignans, flavonoids, phenolic compounds and their glycosides [10–16], were isolated from petroleum ether, ethyl acetate and *n*-butanol extracts of pine needles. The antitumor activity of isolated flavonoids was investigated in greater depth [17]. It is noteworthy that, in addition to the significant anticancer activities of flavonoids, lignans from cedar wood were also reported to show good anticancer activity [18–20].

The current study was herein designed to extract and purify CTL. We set up the methods to analyze the contents of total lignans in CTL as well as the concentrations of honokiol and magnolol in CTL. We systematically investigated the effects of CTL on the proliferation of cancer cells in vitro and revealed a pronounced change in the cell cycle and apoptosis that was possibly required for CTL to exert anti-cancer mechanism.

## Methods

### Plant materials and reagents

The pine needles of *CD* were collected in Lanzhou in June 2014. The plant material was dried in the shade to avoid any destruction of chemical components. Taxonomy identification was performed by Prof. He F. J. in Gansu Province Academy of Medical Science and a voucher specimen has been deposited in the Herbarium for medicinal plants of Gansu Province Academy of Medical Sciences (GSYKY-2014054).

Cell Counting Kit-8 (CCK-8) was purchased from Dojindo Chemical Co. (Japan). RPMI-1640 medium and McCoy’s 5A Medium were obtained from Gibco (USA). DEAE Medium was purchased from HyClone (USA). Penicillin-Streptomycin liquid and Dimethyl sulfoxide (DMSO) were purchased from Solarbio Science & Technology Co., Ltd., (Beijing, China). Fetal bovine serum (FBS) was obtained by Sijiqing Corporation (Hangzhou, China). Methanol (HPLC-grade) was from YuWang Chemical Industry Company (Shan Dong, China). All other reagents and chemicals were of analytical grade.

### Preparation of CTL

The 20~40 mesh powder (10 g) of pine needles of *CD* was extracted with 78% ethanol (20–25 times volume) by means of ethanol hot refluxing for 1.75 h, and then filtered. The filtrate was evaporated to obtain the crude extract (2.18 mg/mL) by using a rotary evaporator. The extract solution was loaded onto column which was wet-packed with AB-8 macroporous resin. The adsorbate-laden column was eluted with 4 BV of deionized water at 1.5 mL/min after adsorption completely, and then with 4 BV of aqueous solution at 1.0 mL/min. The eluting solution was concentrated and dried by rotary evaporator. At boiling water bath, the purified products were heated for 15 min, then rapidly cooled, and diluted to final total 10 mL. The absorbance was estimated by ultraviolet-visible spectrophotometer at 740 nm. The yield of CTL was calculated.

### Determining of lignans in CTL

Holokiol was used as a reference standard to represent the total lignans content. A series of holokiol standard concentrations (22–99 μg·mL^− 1^) were prepared to draw the calibration curve. The holokiol stock solutions were diluted with three times volumes of 5% phosphomolybdic acid. The mixtures were allowed to heat for 15 min at boiling water bath, then to be rapidly cooled and diluted to final total 10 mL with distilled water. The absorbance was estimated at 740 nm by ultraviolet-visible spectrophotometer (Shimadzu Co., Ltd., Japan). The CTL sample was analyzed based on the established calibration curve.

### Cell lines and culture conditions

HeLa, MKN 45, A549, HepG2 and HT-29 cells were purchased from the Shanghai Institute of Cell Biology, Chinese Academy of Sciences (Shanghai, China). A549 cells, HeLa cells, and MKN 45 cells were cultured in RPMI-1640 medium (GIBCO, USA), while HepG2 and HT-29 were cultured in DMEM medium (HyClone, USA). All culture media was incubated at 37 °C in a humidified incubator containing 5% CO_2_, and supplemented with 50 U/mL each of streptomycin and penicillin, 10% FBS.

### Cell viability analysis

Cells (5 × 10^3^) were incubated with different concentrations of CTL or other agents in triplicate in 96-well plates for indicated times at 37 °C in a final volume of 100 μL for 48 h. DMSO (0.1%) was used as vehicle control. The cell viability was assayed by CCK-8 assay according to the manufacturer’s instructions. Briefly, 10 μL of CCK-8 reagent was added to each cluster well and incubated for 2 h at 37 °C. The absorbance was determined at 450 nm using a microplate reader (X-mark, Bio-Rad Laboratories Inc.). Data are calculated as cell viability (% control) and corresponds to the percentage viable cells compared to untreated cells.

### Cell cycle analysis

A549 cells (1 × 10^6^) were plated into 60 mm plates and allowed to adhere for 24 h. The cells then were incubated with different concentrations of CTL (0, 10, 20, 40 μg/mL) for 48 h before digestion with trypsin. The cell suspension was centrifuged at 1500 rpm/min for 5 min. The cells were harvested and washed twice with PBS, fixed with 70% cold ethanol for 24 h at 4 °C, added 200 μL of EDTA (0.1 mM), supplemented with 1 μL of RNaseA (10 mg/mL) for 30 min at room temperature, added 35 μL of 2% tritonx-100, then added 114 μL of PI (50 μg/mL) and incubated in the dark for 15–20 min. The cell samples were placed in Falcon tubes and evaluated by a FACSVerse Calibur flow cytometry.

### Apoptosis assays

A549 cells (1 × 10^5^) were seeded in 6-well culture clusters. After 48 h treatment with different concentrations of CTL, the cells were digested with trypsin and collected. An Annexin V-FITC/PI double staining method was carried out according to the manufacturer’s instruction. Briefly, the cells were washed twice with cold PBS and centrifuged for 5 min at 1500 rpm/min. The cells were suspended in 100 μL of Binding buffer and were incubated with Annexin V-FITC (5 μL) and PI (5 μL) in the dark at room temperature for 15 min, then added 400 μL of Binding buffer, and the apoptotic cells were assayed by a FACS Calibur flow cytometry.

### Statistical analysis

Statistical analysis was conducted by using SPSS 16.0 software. All data were presented as mean ± SD from 3 to 5 different experiments. Statistical differences between groups were analyzed by one-way analysis of variance (ANOVA) and Student’s t-test with statistical significance. A *p* value < 0.05 was used as the criterion for statistical significance.

## Results

### Determination of total lignans in CTL

To assess the total lignans content in CTL, we employed honokiol as the calibration standard, the concentration of total lignan was calculated. When the total lignans content in CTL ranged from 22.0 μg/mL to 99.0 μg/mL, we obtained a good linear relationship. The regression equation was: y = 12.416x-0.1777 (R^2^ = 0.9983), where R, x and y represented the regression coefficient, the concentration of total lignans content in CTL (mg/mL), and the absorbance at 740 nm respectively. By calculating the results, we found that the content of total lignans content in CTL was about 55.77% by using this method.

### Anti-proliferative activities of CTL on different cancer cell lines

**Fig. 1:**
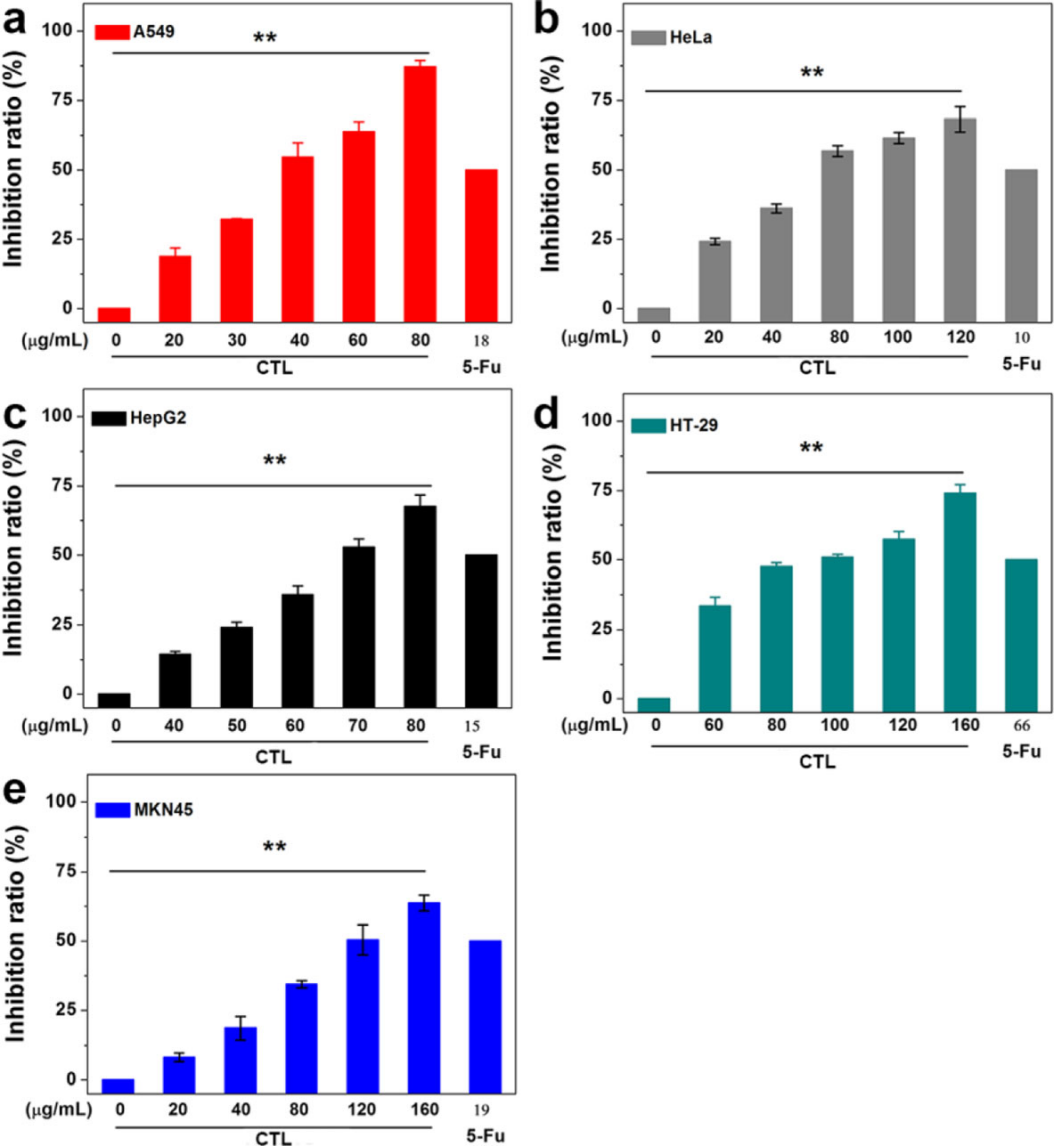
Cytotoxicity of of total lignans from pine needle of CD on five human cancer cell lines. **a** Human lung cancer A549 cells. **b** Human cervical carcinoma HeLa cells. **c** Human hepatocellular carcinoma HepG2. **d** Human colon carcinoma HT-29 cells. **e** Human gastric cancer MKN45 cell

HeLa, MKN 45, A549, HepG2 and HT-29 cells were used to investigate the anti-proliferative activities of CTL. As shown in Fig. 1, after treatment for 48 h, the CTL showed significant inhibition of cell proliferation on five tumor cells in a dose-dependent fashion. The inhibitory effects of CTL were A549 > HeLa > HepG2 > HT-29 > MKN45, with the IC_50_ values of CTL were as follows 39.82 ± 1.74, 62.01 ± 1.37, 67.67 ± 2.64, 99.17 ± 1.67 and 115.84 ± 2.08 μg/mL. The data demonstrated that CTL (0–80 μg/mL) inhibited the proliferation of A549 cells in a dose-dependent fashion (Fig. 1). The CTL displayed a more potent inhibition of A549 cells compared to other tumor cells with the IC_50_ value of 39.82 μg/mL.

### Enhancement of G2/M phase

**Fig. 2:**
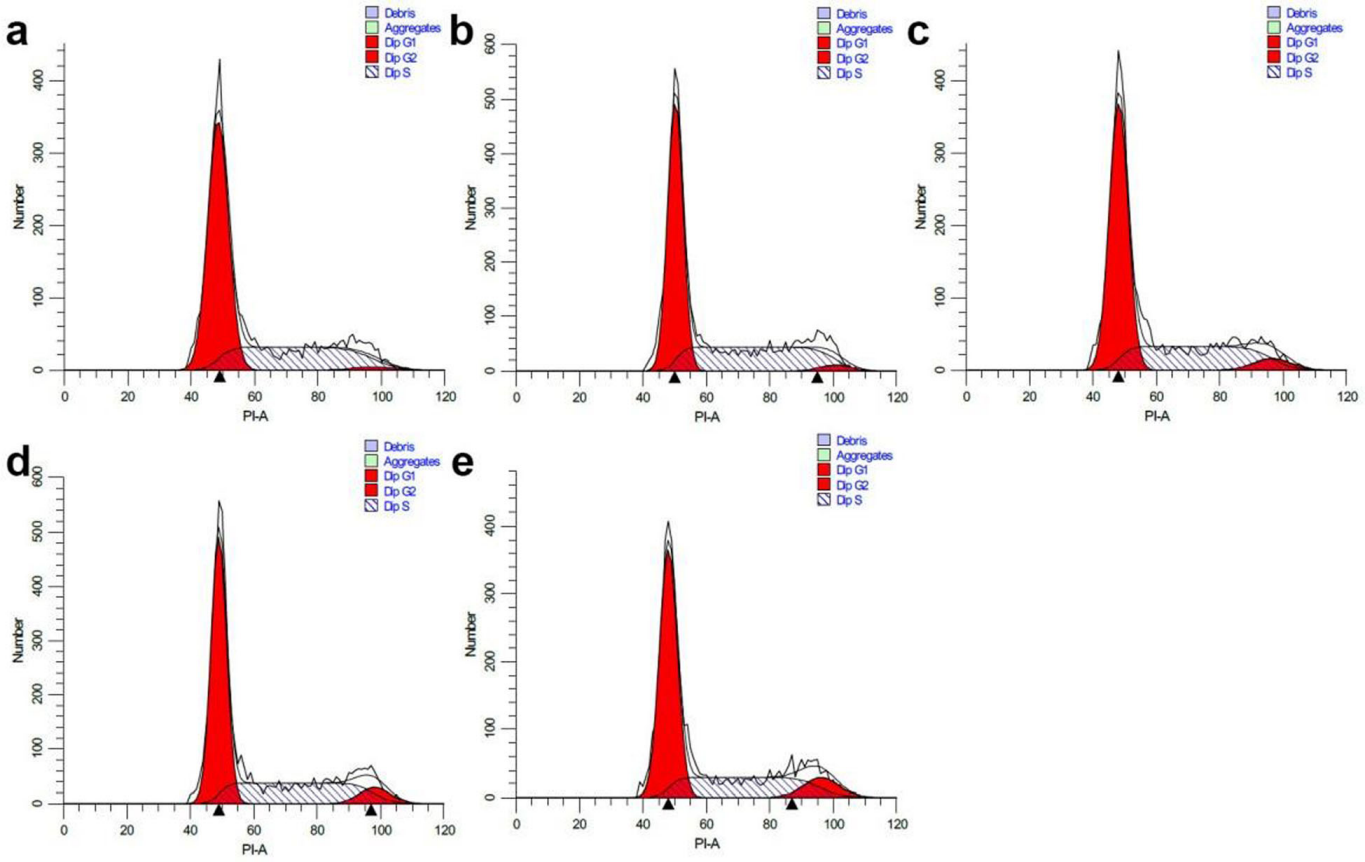
Effects of CTL on the cell cycle of A549 cells. The cells were treated with varying concentrations of CTL (**a-e**: 0, 10, 20, 30 and 40 μg/mL) for 48 h. The A549 cells were stained with propidium iodide (PI) before FACS Calibur flow cytometry analysis

Since the CTL could well inhibit the proliferation of A549 cells, we next investigated the effect of CTL on A549 cell cycle. A549 cells were incubated with varying concentration of CTL for 48 h, assayed by flow cytometric. The data showed that the diploid region appeared obvious in G0/G1 phase, and the proportion of G2 phase increased from 1.51 to 9.40% with the enhancement of CTL concentrations from 0 to 40 μg/mL. Our data indicate that the A549 cells were arrested in G2/M phase (Fig. 2 & Table 1).

**Table 1:** Percentages of A549 cells in S-, G0/G1-,and G2/M-phase (±s, *n* = 3)

Concentration (μg/mL)	Percentages (%)		
	G_0_/G_1_	S	G_2_/M
0	64.26 ± 0.93	34.23 ± 0.32	1.51 ± 0.07
10	56.96 ± 1.73	40.51 ± 0.53	2.53 ± 0.13
20	60.67 ± 1.04	34.10 ± 0.16	5.23 ± 0.74^**^
30	57.82 ± 0.82	35.24 ± 0.36	6.94 ± 0.53^**^
40	58.47 ± 0.33	32.13 ± 0.92	9.40 ± 0.43^**^

**P < 0.01 versus control

### Induction of apoptosis in A549 cells

**Fig. 3:**
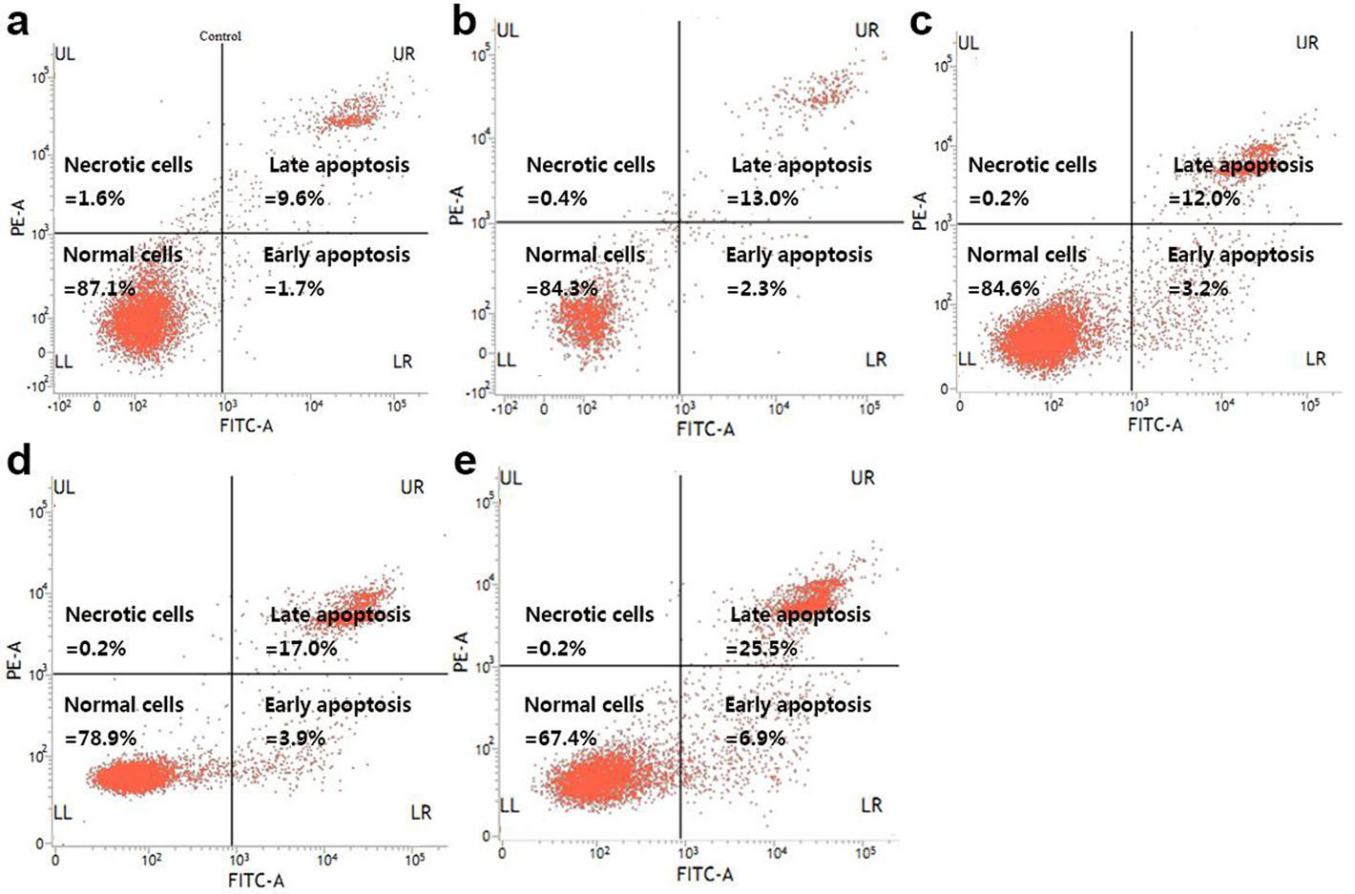
Apoptosis of CTL treatment in A549 cells. The A549 cells were treated with varying concentrations of CTL (**a**-**e**: 0, 10, 20, 30 and 40 μg/mL) for 48 h. Then, the A549 cells were stained with propidium iodide (PI) and Annexin V-FITC as described in Methods. The stained A549 cells were analyzed for apoptosis by using a FACS Calibur flow cytometry

To quantify the apoptotic population, we employed the Annexin V-FITC/PI double staining assay by flow cytometer. As shown in Fig. 3, the late apoptotic cells were positive for PI and Annexin V (the upper right quadrant). The necrotic cell population was negative for Annexin V and positive for PI (the lower right quadrant). The early apoptotic cells were negative for PI but positive for Annexin V (the upper left quadrant). The data demonstrated that the amount of both early and late apoptosis of A549 cells was raised after exposure to CTL for 48 h. As shown in Table 2 and Fig. 3, CTL at the concentrations of 10, 20, 30 and 40 μg/mL raised the apoptosis from 11.29 ± 2.21 to 15.29 ± 3.63, 16.92 ± 2.63, 20.50 ± 3.35 and 32.39 ± 1.74, respectively. Our results demonstrated CTL could induce the A549 cell apoptosis.

**Table 2:** The influence of lignans from the pine needles of *CD* on A549 cell apoptosis (${}_{\overline{x}}$ ±*s*, *n* = 3)

Concentration (μg/mL)	Cell apoptosis distribution percentage		
	LR (%)	UR (%)	Apoptosis (%)
0	1.69 ± 0.97	9.60 ± 1.28	11.29 ± 2.21
10	2.25 ± 1.05	13.04 ± 1.96	15.29 ± 3.63^*^
20	3.24 ± 1.94	12.04 ± 0.80	16.92 ± 2.63^**^
40	3.89 ± 0.45^*^	16.61 ± 3.47^**^	20.50 ± 3.35^**^
80	6.94 ± 0.57^**^	25.49 ± 1.31^**^	32.39 ± 1.74^**^

*P < 0.05, **P < 0.01 versus control

## Disscusion

Cancer is one of the leading causes of death and the incidence of cancer is expected to rise in the coming years. Remedies from traditional medicinal plants are of great interest to cancer patients in the hope of overcoming the well-known risk of side effects caused by synthetic chemotherapeutic drugs. Recently, much effort has been made to develop anti-cancer drugs. With the development of modern molecular medicines, an increasing attention has been given to the identification of natural products capable of inhibiting or retarding the progression of different stages of cancer with the low toxicity, safety and cheapness [21].

Lignans are a large group of polyphenols ubiquitously distributed in plants. As the food-derived cancer preventive compounds, they display potential therapeutic benefit in cancer and may be considered as the candidates for chemotherapeutic drugs [22]. Investigation of the clinical use of lignans, such as pinoresinol, lariciresinol, honokiol, magnolol, secoisolariciresinol, and matairesinol might be valuable strategies in the development of novel anticancer drugs [23–26]. The six subclasses of chemical compounds that have been identified in the CD include the essential oils, terpenoids, lignans, flavonoids, organic acids and others, some of which display evident pharmacological activities when tested in animal and cellular experiments, especially anti-cancer activity [1]. Recently, A CD lignan mixture, prepared from the CD by Singh SK et al, has been shown to induce cytotoxicity in human cancer cells within breast, uterus, colon, liver, prostate and nervous system. This mixture contains 9–13% of (−)-matairesinol, 75–59% of (−)-nortrachelogenin and 7–11% of dibenzylbutyrolactol. The inhibition rate ranges from 49 to 95% at the concentration of 100 μg/ml, with the IC_50_ values of 16.4–116.03 μg/ml. The three compounds in the mixture have been implicated to act synergistically to inhibit the cancer cells [27–29]. Mechanistic studies in Molt-4 and HL-60 cell lines have demonstrated that the CD lignan mixture can induce early NO formation, through which aspartic proteases are activated to generate peroxides. The resultant peroxides depolarize the mitochondrial membrane, leading to the cell apoptosis in a mitochondrial-dependent and mitochondrial-independent apoptotic manner [18–20]. In addition, our group also found that the total flavonoids extracted from the pine needles produced a dose-dependent inhibition of HepG2 cell growth, with the IC_50_ value of 114.12 μg/ml [17].

In this study, we extracted and purified the total lignans from pine needles of *CD*. The CTL were extracted by means of ethanol hot refluxing and the content of total lignans in CTL was about 55.77%. Next, we systematically evaluated the biological activity of total lignans against cancer cells. By the CCK-8 assays, CTL inhibited the growth of A549 cells in a dose-dependent fashion, with the IC_50_ values of 39.82 ± 1.74 μg/mL. Our data showed that the total lignans effectively inhibited the proliferation of A549 cells even at the lower doses (Fig. 1).

Evading apoptosis is one of the key mechanisms underlying malignant cells [30]. Therefore, activation of apoptotic pathways in cancer cells is crucial for the treatment of cancer. The means by which herbal products induce apoptosis has become an issue of great interest. We herein revealed that CTL exhibits potent cytotoxic activity on A549 cells. In order to gain insight into the mechanism of anti-tumor effects of CTL, we studied the possible changes in cell cycle and apoptosis. We know that G2/M is important for the entrance of cells into the M phase and is also associated with tumor cell growth and resistance [31]. Our data showed that the proportion of G2 phase increased from 1.51 to 9.40% with the enhancement of CTL concentrations from 0 to 40 μg/mL, and the total lignans was able to retard the A549 cells at G2/M phase (Fig. 2 & Table 1). CTL also inhibited the growth to a less extent in HeLa, HepG2, MKN28 and HT-29 cells.

Consistent with these results, the apoptotic rate of A549 cells was also enhanced by CTL in a dose-dependent manner (Fig. 3). Our data demonstrated CTL could induce the A549 cell apoptosis. Thus, we disclose a novel molecular mechanism underlying the cellular action of CTL, and shed lights in understanding of the medicinal function of *CD* and provide evidence that CTL have an effective inhibitory effect on tumor growth for the first time.

## Conclusions

In conclusion, we have revealed the anti-cancer potential of the CTL by inducing the apoptosis of A549 cells. The mechanism might involve the regulation of G2/M phase and apoptosis, which would shed lights to extensively investigate how the lignans from pine needles of *CD* function in vivo in the future.

## Data Availability

The datasets used and/or analysed during the current study available from the corresponding author on reasonable request.

## Abbreviations

BV: Bed volume
CCK-8: Cell Counting Kit-8
*CD*: *Cedrus deodara* (Roxb.) Loud
CTL: *Cedrus deodara* total lignans from the pine needles
FBS: Fetal bovine serum
